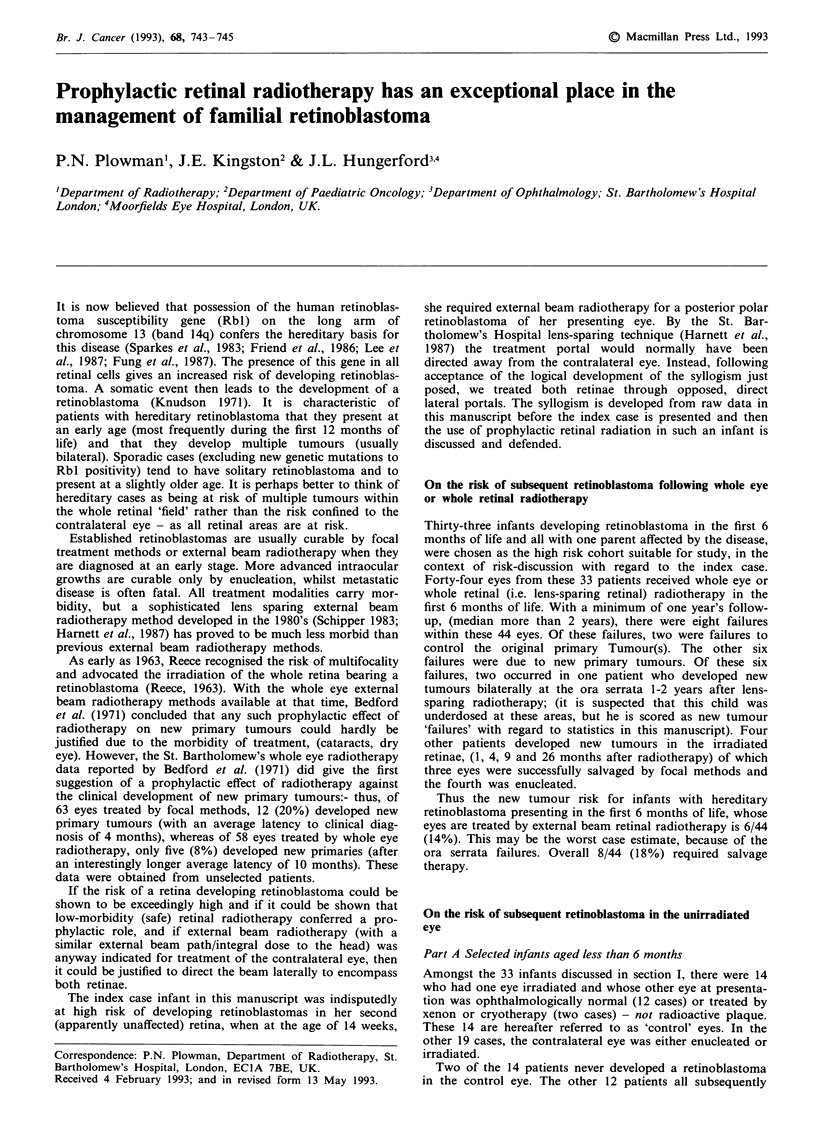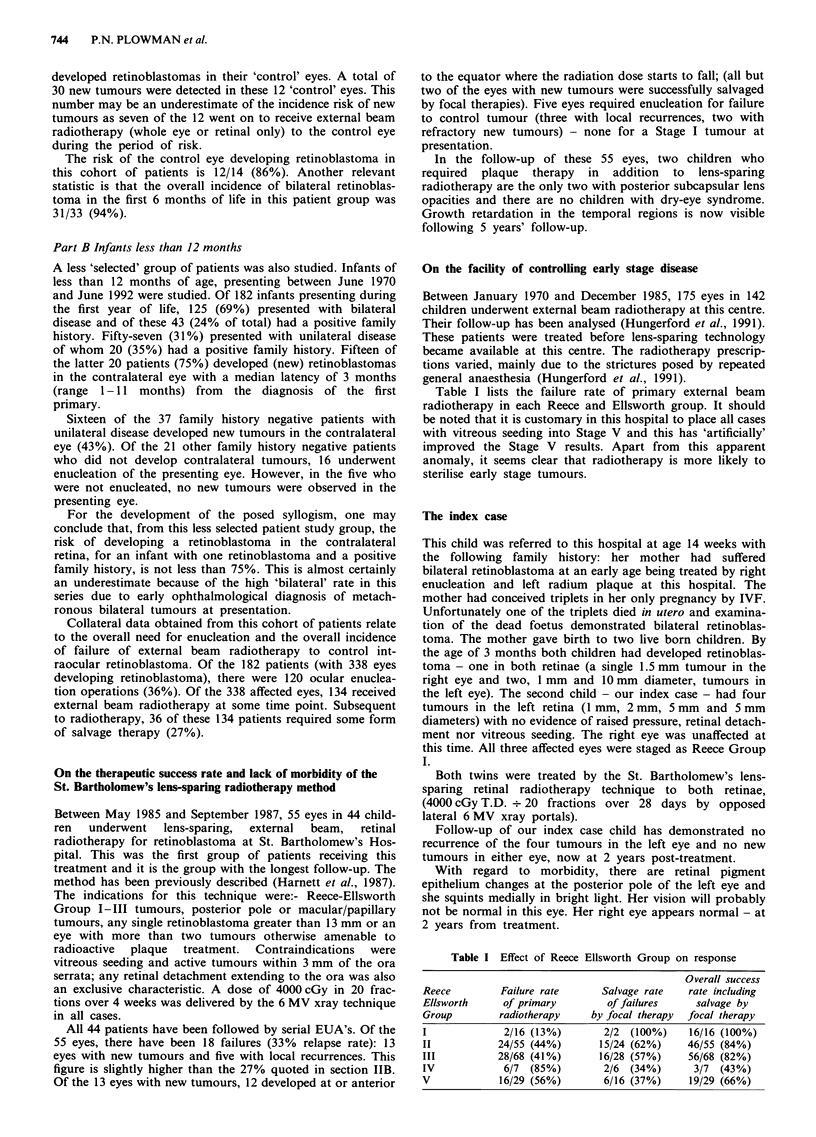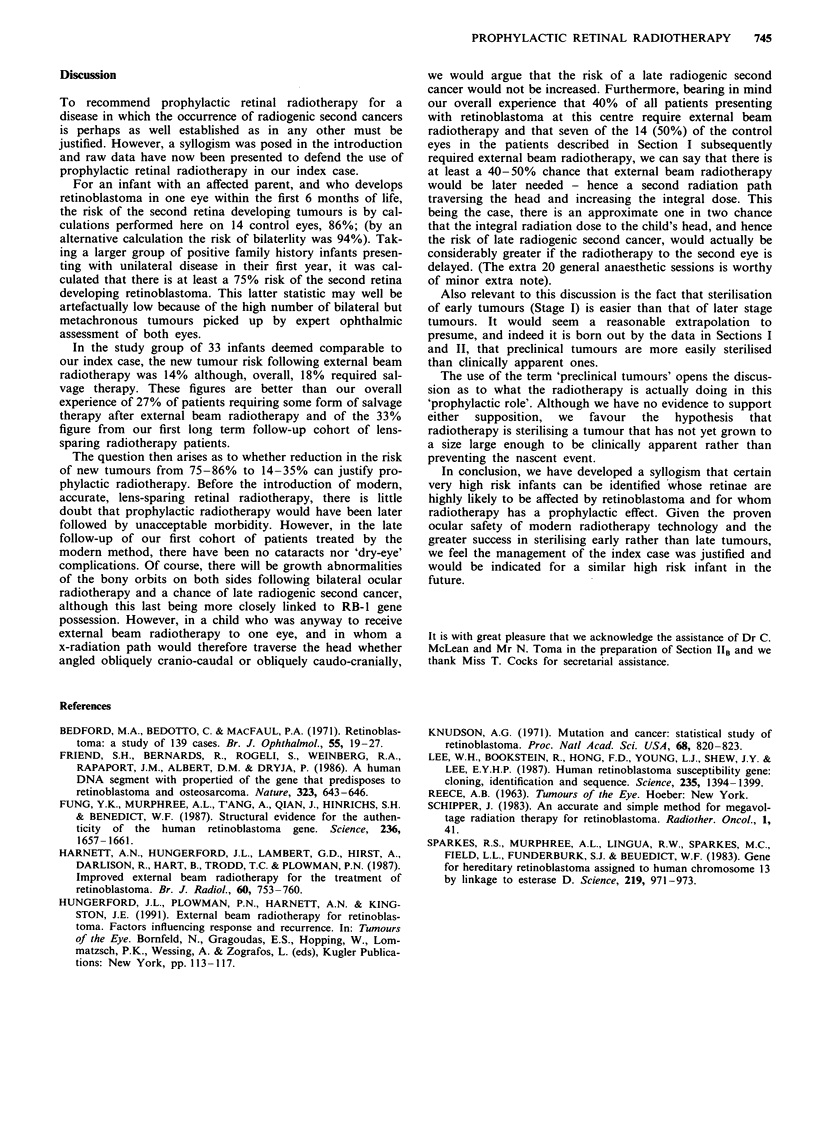# Prophylactic retinal radiotherapy has an exceptional place in the management of familial retinoblastoma.

**DOI:** 10.1038/bjc.1993.421

**Published:** 1993-10

**Authors:** P. N. Plowman, J. E. Kingston, J. L. Hungerford

**Affiliations:** Department of Radiotherapy, St. Bartholomew's Hospital, London, UK.


Br. J. Cancer (1993), 68, 743-745 © Macmillan Press Ltd., 1993

### Prophylactic retinal radiotherapy has an exceptional place in the management of familial retinoblastoma

P.N. Plowman[1], J.E. Kingston[2] & J.L. Hungerford[3,4]

[1]Department of Radiotherapy; [2]Department of Paediatric Oncology; [3]Department of Ophthalmology; St. Bartholomew's Hospital London; [4]Moorfields Eye Hospital, London, UK.

It is now believed that possession of the human retinoblastoma susceptibility gene (Rb1) on the long arm of chromosome 13 (band 14q) confers the hereditary basis for this disease (Sparkes *et al.*, 1983; Friend *et al.*, 1986; Lee *et al.*, 1987; Fung *et al.*, 1987). The presence of this gene in all retinal cells gives an increased risk of developing retinoblastoma. A somatic event then leads to the development of a retinoblastoma (Knudson 1971). It is characteristic of patients with hereditary retinoblastoma that they present at an early age (most frequently during the first 12 months of life) and that they develop multiple tumours (usually bilateral). Sporadic cases (excluding new genetic mutations to Rb1 positivity) tend to have solitary retinoblastoma and to present at a slightly older age. It is perhaps better to think of hereditary cases as being at risk of multiple tumours within the whole retinal 'field' rather than the risk confined to the contralateral eye – as all retinal areas are at risk.

Established retinoblastomas are usually curable by focal treatment methods or external beam radiotherapy when they are diagnosed at an early stage. More advanced intraocular growths are curable only by enucleation, whilst metastatic disease is often fatal. All treatment modalities carry morbidity, but a sophisticated lens sparing external beam radiotherapy method developed in the 1980's (Schipper 1983; Harnett *et al.*, 1987) has proved to be much less morbid than previous external beam radiotherapy methods.

As early as 1963, Reece recognised the risk of multifocality and advocated the irradiation of the whole retina bearing a retinoblastoma (Reece, 1963). With the whole eye external beam radiotherapy methods available at that time, Bedford *et al.* (1971) concluded that any such prophylactic effect of radiotherapy on new primary tumours could hardly be justified due to the morbidity of treatment, (cataracts, dry eye). However, the St. Bartholomew's whole eye radiotherapy data reported by Bedford *et al.* (1971) did give the first suggestion of a prophylactic effect of radiotherapy against the clinical development of new primary tumours:- thus, of 63 eyes treated by focal methods, 12 (20%) developed new primary tumours (with an average latency to clinical diagnosis of 4 months), whereas of 58 eyes treated by whole eye radiotherapy, only five (8%) developed new primaries (after an interestingly longer average latency of 10 months). These data were obtained from unselected patients.

If the risk of a retina developing retinoblastoma could be shown to be exceedingly high and if it could be shown that low-morbidity (safe) retinal radiotherapy conferred a prophylactic role, and if external beam radiotherapy (with a similar external beam path/integral dose to the head) was anyway indicated for treatment of the contralateral eye, then it could be justified to direct the beam laterally to encompass both retinae.

The index case infant in this manuscript was indisputedly at high risk of developing retinoblastomas in her second (apparently unaffected) retina, when at the age of 14 weeks, she required external beam radiotherapy for a posterior polar retinoblastoma of her presenting eye. By the St. Bartholomew's Hospital lens-sparing technique (Harnett *et al.*, 1987) the treatment portal would normally have been directed away from the contralateral eye. Instead, following acceptance of the logical development of the syllogism just posed, we treated both retinae through opposed, direct lateral portals. The syllogism is developed from raw data in this manuscript before the index case is presented and then the use of prophylactic retinal radiation in such an infant is discussed and defended.

Correspondence: P.N. Plowman, Department of Radiotherapy, St. Bartholomew's Hospital, London, EC1A 7BE, UK.
Received 4 February 1993; and in revised form 13 May 1993.

### On the risk of subsequent retinoblastoma following whole eye or whole retinal radiotherapy

Thirty-three infants developing retinoblastoma in the first 6 months of life and all with one parent affected by the disease, were chosen as the high risk cohort suitable for study, in the context of risk-discussion with regard to the index case. Forty-four eyes from these 33 patients received whole eye or whole retinal (i.e. lens-sparing retinal) radiotherapy in the first 6 months of life. With a minimum of one year's follow-up, (median more than 2 years), there were eight failures within these 44 eyes. Of these failures, two were failures to control the original primary Tumour(s). The other six failures were due to new primary tumours. Of these six failures, two occurred in one patient who developed new tumours bilaterally at the ora serrata 1-2 years after lens-sparing radiotherapy; (it is suspected that this child was underdosed at these areas, but he is scored as new tumour 'failures' with regard to statistics in this manuscript). Four other patients developed new tumours in the irradiated retinae, (1, 4, 9 and 26 months after radiotherapy) of which three eyes were successfully salvaged by focal methods and the fourth was enucleated.

Thus the new tumour risk for infants with hereditary retinoblastoma presenting in the first 6 months of life, whose eyes are treated by external beam retinal radiotherapy is 6/44 (14%). This may be the worst case estimate, because of the ora serrata failures. Overall 8/44 (18%) required salvage therapy.

### On the risk of subsequent retinoblastoma in the unirradiated eye

### *Part A Selected infants aged less than 6 months*

Amongst the 33 infants discussed in section I, there were 14 who had one eye irradiated and whose other eye at presentation was ophthalmologically normal (12 cases) or treated by xenon or cryotherapy (two cases) – *not* radioactive plaque. These 14 are hereafter referred to as 'control' eyes. In the other 19 cases, the contralateral eye was either enucleated or irradiated.

Two of the 14 patients never developed a retinoblastoma in the control eye. The other 12 patients all subsequently developed retinoblastomas in their 'control' eyes. A total of 30 new tumours were detected in these 12 'control' eyes. This number may be an underestimate of the incidence risk of new tumours as seven of the 12 went on to receive external beam radiotherapy (whole eye or retinal only) to the control eye during the period of risk.

The risk of the control eye developing retinoblastoma in this cohort of patients is 12/14 (86%). Another relevant statistic is that the overall incidence of bilateral retinoblastoma in the first 6 months of life in this patient group was 31/33 (94%).

*Part B Infants less than 12 months*

A less 'selected' group of patients was also studied. Infants of less than 12 months of age, presenting between June 1970 and June 1992 were studied. Of 182 infants presenting during the first year of life, 125 (69%) presented with bilateral disease and of these 43 (24% of total) had a positive family history. Fifty-seven (31%) presented with unilateral disease of whom 20 (35%) had a positive family history. Fifteen of the latter 20 patients (75%) developed (new) retinoblastomas in the contralateral eye with a median latency of 3 months (range 1–11 months) from the diagnosis of the first primary.

Sixteen of the 37 family history negative patients with unilateral disease developed new tumours in the contralateral eye (43%). Of the 21 other family history negative patients who did not develop contralateral tumours, 16 underwent enucleation of the presenting eye. However, in the five who were not enucleated, no new tumours were observed in the presenting eye.

For the development of the posed syllogism, one may conclude that, from this less selected patient study group, the risk of developing a retinoblastoma in the contralateral retina, for an infant with one retinoblastoma and a positive family history, is not less than 75%. This is almost certainly an underestimate because of the high 'bilateral' rate in this series due to early ophthalmological diagnosis of metachronous bilateral tumours at presentation.

Collateral data obtained from this cohort of patients relate to the overall need for enucleation and the overall incidence of failure of external beam radiotherapy to control intraocular retinoblastoma. Of the 182 patients (with 338 eyes developing retinoblastoma), there were 120 ocular enucleation operations (36%). Of the 338 affected eyes, 134 received external beam radiotherapy at some time point. Subsequent to radiotherapy, 36 of these 134 patients required some form of salvage therapy (27%).

**On the therapeutic success rate and lack of morbidity of the St. Bartholomew's lens-sparing radiotherapy method**

Between May 1985 and September 1987, 55 eyes in 44 children underwent lens-sparing, external beam, retinal radiotherapy for retinoblastoma at St. Bartholomew's Hospital. This was the first group of patients receiving this treatment and it is the group with the longest follow-up. The method has been previously described (Harnett *et al.*, 1987). The indications for this technique were:- Reece-Ellsworth Group I–III tumours, posterior pole or macular/papillary tumours, any single retinoblastoma greater than 13 mm or an eye with more than two tumours otherwise amenable to radioactive plaque treatment. Contraindications were vitreous seeding and active tumours within 3 mm of the ora serrata; any retinal detachment extending to the ora was also an exclusive characteristic. A dose of 4000 cGy in 20 fractions over 4 weeks was delivered by the 6 MV xray technique in all cases.

All 44 patients have been followed by serial EUA's. Of the 55 eyes, there have been 18 failures (33% relapse rate): 13 eyes with new tumours and five with local recurrences. This figure is slightly higher than the 27% quoted in section IIB. Of the 13 eyes with new tumours, 12 developed at or anterior to the equator where the radiation dose starts to fall; (all but two of the eyes with new tumours were successfully salvaged by focal therapies). Five eyes required enucleation for failure to control tumour (three with local recurrences, two with refractory new tumours) – none for a Stage I tumour at presentation.

In the follow-up of these 55 eyes, two children who required plaque therapy in addition to lens-sparing radiotherapy are the only two with posterior subcapsular lens opacities and there are no children with dry-eye syndrome. Growth retardation in the temporal regions is now visible following 5 years' follow-up.

**On the facility of controlling early stage disease**

Between January 1970 and December 1985, 175 eyes in 142 children underwent external beam radiotherapy at this centre. Their follow-up has been analysed (Hungerford *et al.*, 1991). These patients were treated before lens-sparing technology became available at this centre. The radiotherapy prescriptions varied, mainly due to the strictures posed by repeated general anaesthesia (Hungerford *et al.*, 1991).

Table I lists the failure rate of primary external beam radiotherapy in each Reece and Ellsworth group. It should be noted that it is customary in this hospital to place all cases with vitreous seeding into Stage V and this has 'artificially' improved the Stage V results. Apart from this apparent anomaly, it seems clear that radiotherapy is more likely to sterilise early stage tumours.

**The index case**

This child was referred to this hospital at age 14 weeks with the following family history: her mother had suffered bilateral retinoblastoma at an early age being treated by right enucleation and left radium plaque at this hospital. The mother had conceived triplets in her only pregnancy by IVF. Unfortunately one of the triplets died *in utero* and examination of the dead foetus demonstrated bilateral retinoblastoma. The mother gave birth to two live born children. By the age of 3 months both children had developed retinoblastoma – one in both retinae (a single 1.5 mm tumour in the right eye and two, 1 mm and 10 mm diameter, tumours in the left eye). The second child – our index case – had four tumours in the left retina (1 mm, 2 mm, 5 mm and 5 mm diameters) with no evidence of raised pressure, retinal detachment nor vitreous seeding. The right eye was unaffected at this time. All three affected eyes were staged as Reece Group I.

Both twins were treated by the St. Bartholomew's lens-sparing retinal radiotherapy technique to both retinae, (4000 cGy T.D. ÷ 20 fractions over 28 days by opposed lateral 6 MV xray portals).

Follow-up of our index case child has demonstrated no recurrence of the four tumours in the left eye and no new tumours in either eye, now at 2 years post-treatment.

With regard to morbidity, there are retinal pigment epithelium changes at the posterior pole of the left eye and she squints medially in bright light. Her vision will probably not be normal in this eye. Her right eye appears normal – at 2 years from treatment.

**Table I** Effect of Reece Ellsworth Group on response

| *Reece Ellsworth Group* | *Failure rate of primary radiotherapy* | *Salvage rate of failures by focal therapy* | *Overall success rate including salvage by focal therapy* |
|---|---|---|---|
| I | 2/16 (13%) | 2/2 (100%) | 16/16 (100%) |
| II | 24/55 (44%) | 15/24 (62%) | 46/55 (84%) |
| III | 28/68 (41%) | 16/28 (57%) | 56/68 (82%) |
| IV | 6/7 (85%) | 2/6 (34%) | 3/7 (43%) |
| V | 16/29 (56%) | 6/16 (37%) | 19/29 (66%) |

### Discussion

To recommend prophylactic retinal radiotherapy for a disease in which the occurrence of radiogenic second cancers is perhaps as well established as in any other must be justified. However, a syllogism was posed in the introduction and raw data have now been presented to defend the use of prophylactic retinal radiotherapy in our index case.

For an infant with an affected parent, and who develops retinoblastoma in one eye within the first 6 months of life, the risk of the second retina developing tumours is by calculations performed here on 14 control eyes, 86%; (by an alternative calculation the risk of bilaterlity was 94%). Taking a larger group of positive family history infants presenting with unilateral disease in their first year, it was calculated that there is at least a 75% risk of the second retina developing retinoblastoma. This latter statistic may well be artefactually low because of the high number of bilateral but metachronous tumours picked up by expert ophthalmic assessment of both eyes.

In the study group of 33 infants deemed comparable to our index case, the new tumour risk following external beam radiotherapy was 14% although, overall, 18% required salvage therapy. These figures are better than our overall experience of 27% of patients requiring some form of salvage therapy after external beam radiotherapy and of the 33% figure from our first long term follow-up cohort of lens-sparing radiotherapy patients.

The question then arises as to whether reduction in the risk of new tumours from 75–86% to 14–35% can justify prophylactic radiotherapy. Before the introduction of modern, accurate, lens-sparing retinal radiotherapy, there is little doubt that prophylactic radiotherapy would have been later followed by unacceptable morbidity. However, in the late follow-up of our first cohort of patients treated by the modern method, there have been no cataracts nor 'dry-eye' complications. Of course, there will be growth abnormalities of the bony orbits on both sides following bilateral ocular radiotherapy and a chance of late radiogenic second cancer, although this last being more closely linked to RB-1 gene possession. However, in a child who was anyway to receive external beam radiotherapy to one eye, and in whom a x-radiation path would therefore traverse the head whether angled obliquely cranio-caudal or obliquely caudo-cranially, we would argue that the risk of a late radiogenic second cancer would not be increased. Furthermore, bearing in mind our overall experience that 40% of all patients presenting with retinoblastoma at this centre require external beam radiotherapy and that seven of the 14 (50%) of the control eyes in the patients described in Section I subsequently required external beam radiotherapy, we can say that there is at least a 40–50% chance that external beam radiotherapy would be later needed – hence a second radiation path traversing the head and increasing the integral dose. This being the case, there is an approximate one in two chance that the integral radiation dose to the child's head, and hence the risk of late radiogenic second cancer, would actually be considerably greater if the radiotherapy to the second eye is delayed. (The extra 20 general anaesthetic sessions is worthy of minor extra note).

Also relevant to this discussion is the fact that sterilisation of early tumours (Stage I) is easier than that of later stage tumours. It would seem a reasonable extrapolation to presume, and indeed it is born out by the data in Sections I and II, that preclinical tumours are more easily sterilised than clinically apparent ones.

The use of the term 'preclinical tumours' opens the discussion as to what the radiotherapy is actually doing in this 'prophylactic role'. Although we have no evidence to support either supposition, we favour the hypothesis that radiotherapy is sterilising a tumour that has not yet grown to a size large enough to be clinically apparent rather than preventing the nascent event.

In conclusion, we have developed a syllogism that certain very high risk infants can be identified whose retinae are highly likely to be affected by retinoblastoma and for whom radiotherapy has a prophylactic effect. Given the proven ocular safety of modern radiotherapy technology and the greater success in sterilising early rather than late tumours, we feel the management of the index case was justified and would be indicated for a similar high risk infant in the future.

It is with great pleasure that we acknowledge the assistance of Dr C. McLean and Mr N. Toma in the preparation of Section $II_B$ and we thank Miss T. Cocks for secretarial assistance.

### References

BEDFORD, M.A., BEDOTTO, C. & MACFAUL, P.A. (1971). Retinoblastoma: a study of 139 cases. *Br. J. Ophthalmol.*, **55**, 19–27.

FRIEND, S.H., BERNARDS, R., ROGELI, S., WEINBERG, R.A., RAPAPORT, J.M., ALBERT, D.M. & DRYJA, P. (1986). A human DNA segment with propertied of the gene that predisposes to retinoblastoma and osteosarcoma. *Nature*, **323**, 643–646.

FUNG, Y.K., MURPHREE, A.L., T'ANG, A., QIAN, J., HINRICHS, S.H. & BENEDICT, W.F. (1987). Structural evidence for the authenticity of the human retinoblastoma gene. *Science*, **236**, 1657–1661.

HARNETT, A.N., HUNGERFORD, J.L., LAMBERT, G.D., HIRST, A., DARLISON, R., HART, B., TRODD, T.C. & PLOWMAN, P.N. (1987). Improved external beam radiotherapy for the treatment of retinoblastoma. *Br. J. Radiol.*, **60**, 753–760.

HUNGERFORD, J.L., PLOWMAN, P.N., HARNETT, A.N. & KINGSTON, J.E. (1991). External beam radiotherapy for retinoblastoma. Factors influencing response and recurrence. In: *Tumours of the Eye*. Bornfeld, N., Gragoudas, E.S., Hopping, W., Lommatzsch, P.K., Wessing, A. & Zografos, L. (eds), Kugler Publications: New York, pp. 113–117.

KNUDSON, A.G. (1971). Mutation and cancer: statistical study of retinoblastoma. *Proc. Natl Acad. Sci. USA*, **68**, 820–823.

LEE, W.H., BOOKSTEIN, R., HONG, F.D., YOUNG, L.J., SHEW, J.Y. & LEE, E.Y.H.P. (1987). Human retinoblastoma susceptibility gene: cloning, identification and sequence. *Science*, **235**, 1394–1399.

REECE, A.B. (1963). *Tumours of the Eye*. Hoeber: New York.

SCHIPPER, J. (1983). An accurate and simple method for megavoltage radiation therapy for retinoblastoma. *Radiother. Oncol.*, **1**, 41.

SPARKES, R.S., MURPHREE, A.L., LINGUA, R.W., SPARKES, M.C., FIELD, L.L., FUNDERBURK, S.J. & BEUEDICT, W.F. (1983). Gene for hereditary retinoblastoma assigned to human chromosome 13 by linkage to esterase D. *Science*, **219**, 971–973.